# Endoscopic removal of a whole ascaris from the colonic lumen

**DOI:** 10.1055/a-2248-0227

**Published:** 2024-02-22

**Authors:** Cheng Guo, Wei Liu, Yijun Wang, Tao Chen, Meidong Xu

**Affiliations:** 1 66324 Endoscopy Center, Department of Gastroenterology, Shanghai East Hospital, Shanghai, China

A 67-year-old previous healthy woman was admitted to our hospital with paroxysmal abdominal colic. She reported that she loved eating raw food. She underwent colonoscopy, which revealed a reddish white, cylindrical ascaris with horizontal stripes wriggling vigorously in the lumen of the ascending colon ( Fig. 1 **a** ). We used forceps to grab the middle of the live ascaris and pull it out of the patient’s body under direct visualization ( Fig. 1 **b** ; Video 1 ). The ascaris was found to be more than 20 cm long ( Fig. 2 ).

**Fig. 1:**
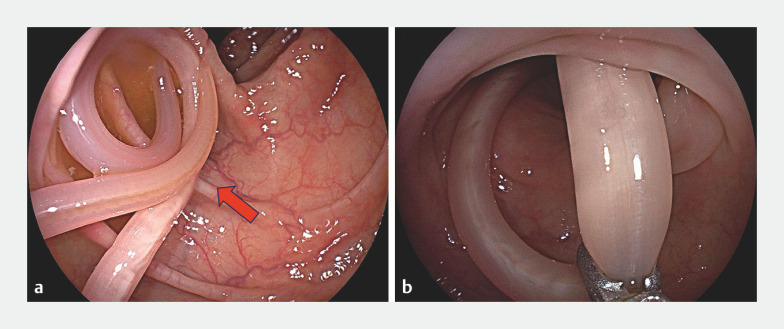
Endoscopic images showing: **a** a live ascaris (red arrow) vigorously wriggling in the lumen of the ascending colon; **b** capture of the ascaris using forceps.

Colonoscopy revealing a live ascaris within the ascending colon, and its endoscopic removal.Video 1

**Fig. 2:**
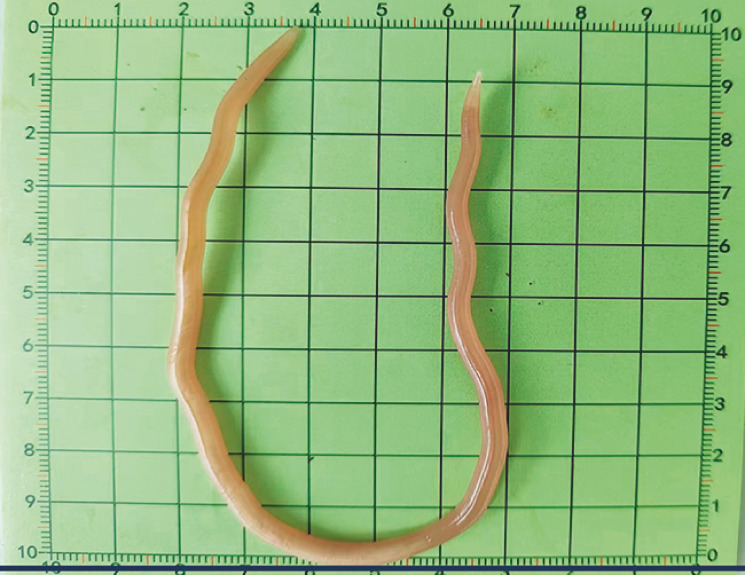
Photograph of the 20-cm ascaris that was pulled alive from the colonic lumen.

Immediately following the procedure, the patient's abdominal pain was significantly reduced. Antiparasitic treatment, in conjunction with education of the patient and family about hygiene and sanitation, were subsequently introduced.

Ascaris is one of the nematodes with the highest incidence in developing areas. It can parasitize any part of the digestive tract, especially the jejunum and proximal ileum, but is rarely found in the colon. Ascaris can lead to abdominal pain, cholangitis, obstructive jaundice, pancreatitis, and gallstones 1 . Antiparasitic treatment, and improved hygiene and sanitation are important in preventing the spread of disease. In this case, the live ascaris was the cause of the patient’s abdominal pain and was removed under direct visualization.

Endoscopy_UCTN_Code_CCL_1AD_2AJ

## Notes

Endoscopy E-Videos https://eref.thieme.de/e-videos
*E-Videos* is an open access online section of the journal *Endoscopy* , reporting on interesting cases and new techniques in gastroenterological endoscopy. All papers include a high-quality video and are published with a Creative Commons CC-BY license. Endoscopy E-Videos qualify for HINARI discounts and waivers and eligibility is automatically checked during the submission process. We grant 100% waivers to articles whose corresponding authors are based in Group A countries and 50% waivers to those who are based in Group B countries as classified by Research4Life (see: https://www.research4life.org/access/eligibility/ ). This section has its own submission website at https://mc.manuscriptcentral.com/e-videos .
